# Seed Potato Bacteria Transfer Across Generations Within the Tuber Flesh

**DOI:** 10.1007/s00248-026-02758-7

**Published:** 2026-04-09

**Authors:** Sumitra Saha, Anish S. Shah, Penghao Wang, Treena I. Burgess, Kirsty L. Bayliss

**Affiliations:** 1 Centre for Crop and Food Innovation, Food Futures Institute, and School of Agricultural Sciences, Murdoch University, Murdoch, 6150 Australia; 2 Department of Biotechnology, Bangladesh Agricultural University, Mymensingh, 2202 Bangladesh; 3 School of Medical, Molecular and Forensic Sciences, Murdoch University, Murdoch, 6150 Australia; 4 Cooper Medical School of Rowan University, Camden, NJ 08103 USA; 5 Harry Butler Institute, Murdoch University, Murdoch, 6150 Australia

**Keywords:** Microbiome, Generational transfer, Compartments, Microbiome resilience, Vertical transfer consistency, Microbial selection

## Abstract

Potato crops are susceptible to pathogens and environmental extremes. Microbiomes support plant health and stress tolerance, and microbes can transfer across generations in vegetatively propagated potatoes. However, the extent and functional relevance of this transfer are poorly understood. This study investigated bacterial transfer across three tuber generations, from seed to granddaughter in two potato cultivars, Nadine and Royal Blue. Bacterial communities in the peel and flesh compartments were sequenced. The granddaughter generation was cultivated in two separate fields to determine the consistency of vertical transfer, and the tare soil bacterial community was a proxy for environmental acquisition. The overall community composition was influenced by generation, compartment, cultivar and field. Horizontal acquisition significantly increased across generations and was the primary source, accounting for more than 98% of the granddaughter tuber bacteria. Peel had a significantly higher number of horizontally acquired ASVs than flesh. Only a small set of seed tuber bacteria ASVs were vertically transferred to the granddaughter tubers. The overall vertical transfer probability was 1.8% across compartments, cultivars, and fields, and it was higher in flesh than in peel. Cultivar-specific probabilities were 1.8% for Nadine and 1.5% for Royal Blue. Field variance was minimal, indicating consistent vertical transfer regardless of where the tubers were grown. Taxa with stable vertical transfer included Streptomycetaceae, Xanthobacteraceae, Devosiaceae, Sphingomonadaceae, and Micrococcaceae. Vertically transferred ASVs were predicted to have functions associated with core metabolic and stress response pathways. This study confirmed consistent vertical transfer of bacteria across potato tuber generations, mainly in the flesh.

**Supplementary Information:**

The online version contains supplementary material available at 10.1007/s00248-026-02758-7.

## Introduction

The potato (*Solanum tuberosum*) is an important vegetable crop consumed by over two-thirds of the world’s population. It is ranked the third most important staple crop worldwide due to its high yield and efficient resource use [1]. However, potato production is challenged by biotic and abiotic stress, such as heat stress and drought [2, 3]. Traditional breeding and genetic approaches to improve stress resilience are often slow, labour-intensive, and struggle to keep pace with rapidly changing environmental conditions [4]. These limitations have increased interest in more sustainable strategies, such as utilising the potato microbiome, which already coexists with the plant, to improve stress resilience by natural means [5]. For example, beneficial bacterial taxa associated with potato crops could be incorporated into microbiome-assisted breeding programs, developed as bioinoculants or biofertilisers, or adopted in management practices to support crop growth and tolerance to abiotic and biotic stresses. The plant microbiome is the diverse microbial community that colonises internal and external plant tissues and contributes to plant health and ecosystem functioning. Plants acquire these microbes either horizontally from environmental sources such as soil or vertically through seeds [6]. In vegetatively propagated crops, such as potatoes, microbes are vertically transferred from seed tubers to daughter tubers via stolons, potentially maintaining microbial families across generations [7, 8]. The soil attached to the tuber (tare soil) contributes additional microbial communities [9]. Beneficial bacteria applied to seed tubers successfully colonised the roots and rhizospheres of the growing plants [10], further supporting the horizontal acquisition of microbes, and inoculation as a potential resource for improving potato growth and health.

The potato tuber peel and flesh each host their own microbial community [8]. The peel is in direct contact with the soil and is vulnerable to changes in the surrounding environment. The flesh is protected inside the tuber and likely supports a more stable microbiome. Peel and flesh differences were reported in field-grown tubers [8]; however, only the vertical transfer of field-unique microbes (rather than all seed tuber communities) to daughter tubers was measured, irrespective of compartment. A pot trial examined several compartments of seed tubers without considering them as sources for vertical transfer [7]. The transfer of seed tuber bacteria to the next generation in the field and their association with different tuber compartments has not been fully investigated. No study has examined the function of transferred bacteria in potatoes. These gaps limit understanding of why certain bacteria are transferred through the tuber and how stable this transfer is across generations in different compartments. Host genetics are likely to contribute to the assembly of these vertically transferred communities, consistent with the concept of the plant-dominated microbiome [11]. This concept suggests that breeding could be used to manipulate the composition and stability of the beneficial microbiome across generations. Knowledge of compartment-specific vertical transfer may therefore inform breeding strategies to select potato cultivars with beneficial bacteria, to improve crop resilience.

In this study, the vertical transfer of potato seed tuber bacterial communities was investigated across three generations in the peel and flesh of two cultivars. Bacteria were selected as they occur in potato peel and flesh, and have well established roles in plant health, productivity and stress tolerance [12–14]. Bacterial communities are also sensitive to environmental and plant factors such as soil type, location, and genotype, which makes them suitable indicators to measure vertical transfer and horizontal acquisition. Although fungal endophytes may also be transferred across generations in potato compartments, their sequencing and interpretation are often complicated by co-amplification of host DNA [15]. For this reason, the present study focused on bacteria as a reliable starting point to investigate vertical transfer in tuber compartments. It was hypothesised that tuber bacterial communities would differ between compartments, cultivars, and fields; that only a limited number of seed tuber bacteria, potentially linked to plant health and stress resilience, would be vertically transferred across generations; and that this transfer would differ between the peel and flesh. The tare soil bacterial communities of granddaughter tubers from two fields were used to determine the extent of horizontal acquisition.

## Materials and Pethods

### Seed Potatoes

Certified seed potatoes (3rd generation, G3) of two genetically distinct cultivars, ‘Nadine’ and ‘Royal Blue’ [16], were purchased from the accredited supplier GP Ayres and Sons in Albany, Western Australia (WA) (Table S1). The seed lot codes were 23AY101(Nadine) and 23AY103 (Royal Blue); R/A numbers: 71,494 (Nadine) and 71,943 (Royal Blue); harvested in April 2023.

### Daughter Tuber Trial - Location, Preparation and Planting

Before planting, seed potatoes were allowed to green sprout for 10–15 days. The tubers were arranged in single layers on an open bench in a well-lit glasshouse at 22–25 °C to promote uniform sprout development. Seed potatoes were planted at Brunswick Junction, WA (33.25°S, 115.83°E) to obtain daughter tubers (Fig. S1). Temperature and relative humidity data were obtained from the nearest weather station (https://weather.agric.wa.gov.au/station/BJ). In the season prior, the plot was used to grow brassica crops. The plot was cleared of debris, weeds, and rocks, and the soil was ploughed to a depth of 15–20 cm. The seedbeds were elevated to facilitate soil drainage.

Both cultivars were planted on 13/11/2023 in a 10 m × 6 m plot in two rows with a 20 cm spacing between individual plants and a 2 m spacing between rows. Buffer zones of 2 m were maintained at the top and bottom of the plot and along both plot edges. The R package agricolae was used to randomise both the cultivars in each row (Fig. S2). The seed potatoes were planted at a depth of 8–10 cm, and a layer of meadow hay silage was applied as mulch for weed control and to maintain soil moisture. The soil was mounded periodically around the base of the emerging plants to encourage tuber formation along the stolons. The plot was irrigated using Netafim drip lines at a flow rate of 8 L h⁻¹. Irrigation was adjusted to maintain adequate soil moisture throughout the growing period; while the exact duration and frequency of watering events were not recorded, the system was operated as needed to prevent visible plant wilting. No synthetic fertilisers were applied as the farm follows organic farming practices and had previously been used for dairy cow grazing and had rich, fertile soil (D. Doepel, pers. comm.). Daughter tubers were harvested 102 days after planting when potato plants reached senescence, indicated by leaf yellowing and canopy collapse. Tubers were collected in hessian bags after harvesting. After collecting samples, the remaining tubers were stored at 4 °C in hessian bags for the next trial.

### Granddaughter Tuber Trial – Location, Preparation and Planting

To assess the stability and consistency of microbial transfer from seed tubers, daughter tubers were planted in the following season at the same location but in a different plot on 15/8/2024 and also in a new plot at Murdoch University (32.07°S, 115.84°E), WA, on 17/6/2024. This study strictly avoided continuous potato cropping. The plot at Brunswick used for the granddaughter trial was rotated with zucchini before potato planting and the Murdoch plot was previously fallow pasture. Both sites were cleared of weeds and large stones as described above. To prepare the Murdoch site, Wandalup compost (C-Wise Western Australia, Table S2) was applied at a depth of 5 cm and incorporated into the soil by ploughing to a depth of 15–20 cm, then allowed to settle for two months before planting. Climatic data for Murdoch was obtained from the Murdoch University Weather Station, accessible at http://wwwmet.murdoch.edu.au/.

On planting day, soil samples were collected from each site using the systematic unaligned grid soil sampling method to assess available nutrients and pH [17]. Each experimental plot was subdivided into five 10 m² grids, with sampling points positioned in a non-aligned pattern within each grid to avoid spatial bias. Soil samples were collected from a depth of 10–15 cm using a clean trowel. Five samples were combined into a single composite sample and sent for soil physicochemical analysis to CSBP Ltd., Bibra Lake, WA (https://csbp-fertilisers.com.au/services/lab*)* (Table S3). Following green sprouting under well-lit, room-temperature conditions (as described above), daughter tubers were planted at each site using the same plant-to-plant spacing, row spacing, and randomisation scheme previously outlined.

### Agronomic Practices

Agronomic practices at Brunswick Junction followed the protocol described above. At the Murdoch site, mounding was performed as described for Brunswick Junction. Irrigation at Murdoch was applied each afternoon manually using a hose until the soil surface was visibly moist. Only at the Murdoch site, Red fertiliser (EcoGrowth Ltd., Australia) was applied at 50 g m⁻² (equivalent to approximately 20 g per plant, based on a 20 cm plant-to-plant spacing × 2 m row spacing) at planting and was loosely incorporated into the soil. Subsequently, Prime (EcoGrowth Ltd., Australia) was applied at the same rate during the mounding-up stage to promote plant growth and development. These application rates were based on nutrient-removal estimates for a 30 t ha⁻¹ potato crop (D. Sutton, EcoGrowth, pers. comm.), which approximate 97 kg N, 66 kg P, and 112 kg K ha⁻¹. These rates did not account for potential additional efficiencies or losses arising from compost application, nutrient forms, or leaching (Table S4). Forty-nine days after planting, Dipel biological insecticide (Yates, Australia) containing *Bacillus thuringiensis* subsp. Kurstaki (Btk) strain ABTS-351 was applied according to the manufacturer’s directions at both sites to control insect pests, such as caterpillars.

### Tuber and Tare Soil Sample Collection and Processing

Ten tubers were randomly selected at harvest from each cultivar and each generation (seed, daughter, and granddaughter), with each tuber representing an independent replicate (*n* = 10 per generation). Flesh and peel samples were collected from the daughter and granddaughter tubers immediately after harvest. All tubers were thoroughly washed with deionised water prior to sampling. Approximately 5–7 g of peel from each tuber was collected using a sterilised potato peeler, sampling evenly around the tuber surface. To prevent cross-contamination, separate knives, peelers, mortars, and pestles were used for each sample. All tools were cleaned with water, treated with 4% sodium hypochlorite, and sterilised by autoclaving before use. Peel samples were then transferred into 10 mL of 0.85% saline solution (NaCl) in 50 mL tubes and shaken vigorously by hand for 2–3 min to remove adhering soil, as described by Lee, Kim [18].

After peeling, flesh samples weighing 9–10 g were collected using a sterilised knife from the cortex, outer medulla, and inner medulla (Fig. S3). Peel and flesh samples were separately processed into a fine powder using sterilised mortars and pestles in liquid nitrogen. Approximately 0.25 g of each replicate was subsampled into 2 mL microcentrifuge tubes for DNA extraction, and all main and subsamples were stored at − 20 °C until further use.

Ten tare soil samples were also collected from the same granddaughter tubers prior to peeling and washing. Approximately 2–5 g of soil adhering to the tuber surface was gently rubbed off, collected into 50 mL centrifuge tubes, and stored at − 20 °C until further processing.

### DNA Extraction

Approximately 0.25 g of powdered tuber samples were used for DNA extraction, following the manufacturer’s instructions (Qiagen DNeasy PowerSoil Pro Kit, Cat. No. 47016), with slight modifications. The samples were incubated in the Power-bead tubes for 10 min in the CD1 solution. Subsequently, they underwent lysis in a Qiagen Tissue Lyser II for 2 min at 25 Hz. DNA was eluted from the column after a 5-min incubation in 52 µL of elution buffer. The quantity and quality of the extracted DNA were evaluated using a NanoDrop ND-1000 spectrophotometer. Samples that did not meet the 260/280 absorbance ratio criterion of 1.8–2.0 were discarded, and DNA extraction was repeated. The DNA concentration ranged from 10 to 30 ng/µl for all samples. The DNA samples were stored at − 20 °C until further use.

### Metabarcoding

PCR amplification of the V4 region of the 16 S rRNA gene was performed using the primers 515f [19] and 806r [20]. Amplification was conducted in a 25 µL reaction mix consisting of 12.5 µL of Promega GoTaq^®^ Green Master Mix, 0.5 µL of each forward and reverse primers (final concentration of 0.2 µM), 1.5 µL each of mitochondrial peptide nucleic acid (mPNA) and chloroplast peptide nucleic acid (pPNA) (final concentration of 0.25 µM), 6 µL of PCR-grade water, and 2.5 µL of the template DNA. Mitochondrial and chloroplast DNA amplification was inhibited using mPNA and pPNA clamps, respectively (PNA Bio, USA). The thermal conditions used were an initial denaturation at 94 °C for 3 min, followed by 30 cycles of denaturation at 94 °C for 45 s, annealing at 56 °C for 45 s, and extension at 72 °C for 45 s, with a final extension step at 72 °C for 7 min (Biorad, T100 thermal cycler). Positive (ZymoBIOMICS Microbial Community DNA Standard) and negative (H_2_O with no template DNA) controls were included during PCR amplification and sequencing. Two PCR replicates were included for each sample and pooled after visualising the PCR results on a 1% agarose gel. The pooled PCR products were sent to the Australian Genome Research Facility (Perth, Western Australia) for amplicon purification, indexing, and pooling, followed by sequencing on the Illumina MiSeq 2 × 250 bp PE platform.

### Bioinformatics

All sequence quality control and processing were conducted in R (v4.4.2) using RStudio (v4.3.3). Primer sequences were identified and removed using Cutadapt (v4.0) [21]. Quality filtering, denoising, chimera removal, read pair merging and ASV inference were performed with the DADA2 pipeline (v1.22) [22]. Reads were filtered to exclude low-quality bases, with a maximum expected error rate of two for both forward and reverse reads. Following denoising, paired reads were aligned and clustered into Amplicon Sequence Variants (ASVs). After removing the chimera, approximately 99.1% of the original sequences remained. Subsequent filtering of chloroplast and mitochondrial sequences reduced the processed reads to 69% of the initial reads. The remaining reads were assigned to bacterial taxa using the SILVA reference database (v138.2) [23]. ASVs present in only a few samples or classified as non-bacterial taxa (e.g., archaea) were filtered, accounting for 0.21% of the remaining reads after the initial filtering steps. The final dataset contained 3,535,360 reads and 19,006 ASVs. The processed dataset, including taxonomic classifications and sample metadata, was integrated into the phyloseq package (v1.44.0) [24] for downstream statistical analysis. Taxonomic aggregation was performed at multiple levels, from ASV to genus, family, and phylum, with family-level data selected for downstream analyses to preserve biological distances between samples. Taxa with missing family-level annotations were manually reassigned as non-classified family_order names (e.g., NcF_Bacillaceae) following the approach described by Addison, Rúa [25].

### Statistical Analyses

All statistical analyses were performed in R using RStudio. Data exploration included outlier detection and normality assessment using the Shapiro–Wilk test [26]. Data visualisations were generated using the ggplot2 package (v4.4.1), and statistical significance was defined as false discovery rate (FDR) adjusted *P* < 0.05 unless otherwise specified.

Library size distributions were examined prior to normalisation. Library sizes differed between compartments but were consistent within cultivar. Rarefying the full dataset would have removed a large proportion of reads, so cumulative sum scaling was applied. Peel and flesh richness, Shannon diversity and Pielou evenness were calculated for each cultivar. Shannon diversity was compared across generations within each compartment and cultivar using one-way analysis of variance with Tukey post hoc tests. Richness and evenness were analysed with Kruskal-Wallis tests followed by Dunn pairwise comparisons with Benjamini-Hochberg correction. Results are presented as mean ± standard error. Bray–Curtis dissimilarities were calculated from CSS-normalised data using the phyloseq package and visualised using principal coordinate analysis (PCoA) in ggplot2 [27]. Community differences across generations (seed, daughter, granddaughter), compartments (peel, flesh, tare soil), cultivars, and field sites were evaluated using PERMANOVA (999 permutations) [28] and pairwise permutation tests [29].

Unique and shared ASVs across generations and granddaughter compartments were visualised with JVenn [30]. The percentage of seed ASVs that moved from seed to daughter and granddaughter tubers was shown with Sankey diagrams produced with the ggalluvial package [31]. For each cultivar, compartment and generation, ASVs present in the seed and transferred to later generations were classified as vertically transferred, and ASVs present only in later generations were classified as horizontally acquired. For each generation, the vertical percentage was calculated as the number of seed-tuber ASVs divided by the total ASVs present in that generation. The horizontal percentage was calculated as the number of newly acquired ASVs divided by the total ASVs in that generation. Differences between vertically transferred and horizontally acquired ASVs within each generation transition were assessed using paired Wilcoxon tests, with post hoc comparisons adjusted using the Benjamini–Hochberg method. Differences in horizontally acquired and vertically transferred ASVs between peel and flesh were assessed using a negative binomial mixed model that included field, compartment and generation transition as fixed effects, with cultivar included as a random effect [32].

Vertical transfer probabilities were estimated from ASV counts using generalised linear mixed models (GLMMs) with binomial error structures, fitted with lme4 and glmmTMB [32, 33] to account for the proportional response and the hierarchical structure of the data defined by compartment, cultivar, and field. Models quantified the proportion of seed ASVs detected in the next generation by the total number of ASVs present in the seed generation. Cultivar and compartment were fitted as fixed effects, and field was included as a random intercept when supported by model fit criteria. The significance of fixed terms was evaluated using Wald χ² tests, and estimated marginal means were obtained on the response scale using the emmeans package. Model diagnostics and residual checks were performed using DHARM [34]. When overdispersion or convergence issues occurred, models were re-fitted using either an observation-level random effect, bias-reduced estimation (brglm2) or cluster-robust covariance estimators (sandwich, lmtest). The ASVs that were transferred across all generations were aggregated at the family level to obtain relative abundances within each Generation × Cultivar × Compartment combination. Family-level relative abundances were compared across generations within each cultivar using Kruskal-Wallis tests, followed by Dunn’s post hoc test with P values adjusted for multiple testing using the Benjamini-Hochberg method.

Broad-sense heritability (H²) was used as a statistical measure to quantify the proportion of variance in ASV abundance explained by generation, reflecting the generational consistency of each ASV. In this context, H² does not refer to genetic inheritance or host genetic control. H^2^ was estimated for seed ASVs transferred across generations within each cultivar–compartment combination to evaluate the reliability of vertical microbial transfer. Here, H² Abundance data were log10^(Abundance+1 × 10−6)^ transformed and fitted using the lme4 package, treating generation as a random effect, according to Walters, Jin [35]. For each ASV, the following model was fit independently within each cultivar and compartment:

$$\mathrm{Abundance}\;\mathrm{ijk}=\mathrm\mu+\mathrm{Gi}+\mathrm{\epsilon ijk}$$

Where µ is the overall mean, Gi is the random effect of the 𝑖th generation, and εijk is the residual error. Broad-sense heritability was then calculated as:

$$\mathrm H^2=\mathrm{VG}/\left(\mathrm{VG}+\mathrm{VE}\right)$$

Where VG is the variance component for generation and VE the residual variance.

The statistical significance of the observed heritability estimates was assessed via 999 random permutations of the generation labels. ASVs with empirical *P* < 0.05 were considered statistically consistent across generations. Observed and permuted H² distributions were visualised with violin plots, ASVs labelled by taxonomic family (numeric suffixes, e.g., Family_1, Family_2, added if multiple per family).

Predicted functions of vertically transferred bacteria within potato tuber compartments are limited, so were assessed using the Phylogenetic Investigation of Communities by Reconstruction of Unobserved States (PICRUSt2) pipeline (Supplementary data). No direct functional activity was measured.

## Results

### Overall Bacterial Diversity, Community Composition, and Relative Abundance in Potato Tubers and Tare Soil

Mean richness, Shannon diversity and evenness increased from seed to daughter and granddaughter tubers in both flesh and peel in Nadine and Royal Blue in both fields (Table S5). Seed tubers differed significantly (*P* < 0.0001) from daughter and granddaughter tubers in all pairwise comparisons (Table S6). The only comparison that was not significant (*P* > 0.05) was Royal Blue flesh, where the Brunswick and Murdoch granddaughter tubers did not differ.

**Fig. 1:**
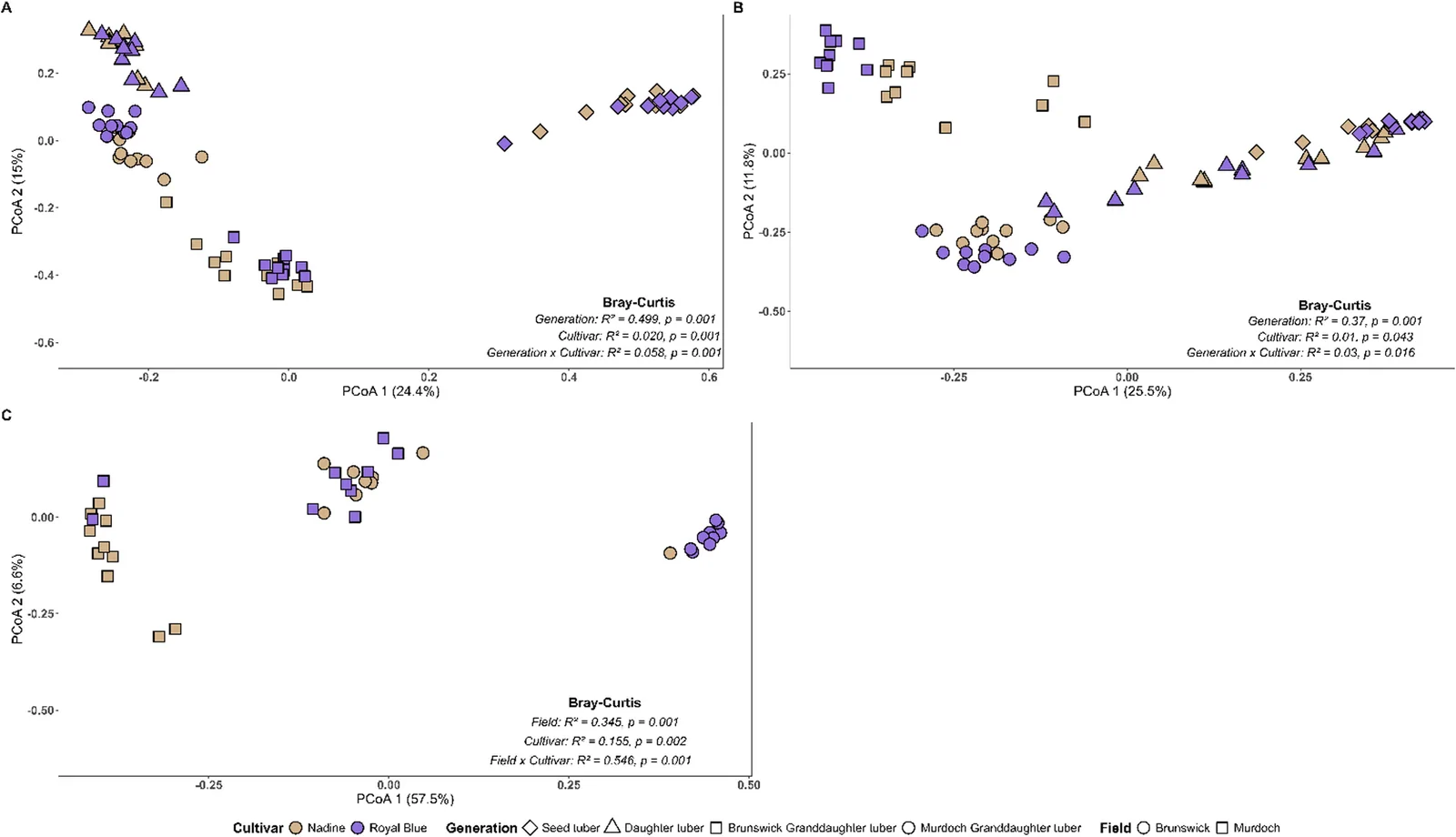
Principal Coordinate Analysis of bacterial community composition in the peel (**A**) and flesh (**B**) of seed, daughter, and granddaughter tubers of potato cultivars Nadine and Royal Blue, grown at two Western Australian farms, and tare soil (**C**) associated with granddaughter tubers of the same cultivars

Generation was the primary driver of bacterial community variation, with a small but consistent interaction with cultivar. Generation explained most of the community variation, accounting for 37% in the flesh and 49% in the peel (*P* = 0.001 for both) (Fig. 1). In the flesh, the cultivar contributed to 1% of the variation, while the interaction with generation accounted for 3% (*P* = 0.001) (Fig. 1A). Flesh communities from seed and daughter tubers clustered closely in the PCoA ordination, indicating similar community structures in those generations (Fig. 1A). In the peel, cultivar explained 2% of the variation (*P* < 0.001), and the interaction with generation explained 5% (*P* < 0.001) (Fig. 1B). Peel communities formed distinct generational clusters with strong separation between seed and daughter tubers. For the granddaughter tuber tare soil, the field explained 34% of the bacterial community variation (*P* = 0.001), and cultivar explained 15% (*P* = 0.002) (Fig. 1C). The cultivar-field interaction explained 54% of the variation (*P* = 0.001), indicating that the two factors did not act independently (1C).

Compartments and cultivars differed in taxonomic composition. Sandaracinaceae were absent from seed tubers but were significantly (*P* < 0.001) abundant in daughter and granddaughter tubers of both cultivars and in both compartments (Table S7, Figs. S4 and S5). In the flesh of both cultivars, Bacillaceae and Caldalkalibacillaceae had higher relative abundance than other families across all generations (Fig. S4 and S6). Nadine flesh had a higher abundance (*P* < 0.001) of Polyangiaceae and Caldalkalibacillaceae than Royal Blue, while Royal Blue flesh had higher Bacillaceae than Nadine. In the peel, Bacillaceae and Caldalkalibacillaceae had higher relative abundance in Royal Blue than in Nadine (*P* < 0.05) (Fig. S5 and S6). Royal Blue peel also had higher relative abundances of Comamonadaceae and Sandaracinaceae than Nadine (*P* < 0.001).

Granddaughter peel communities had the greatest variability (*P* < 0.05), and there was a clear difference between Murdoch- and Brunswick-grown tubers. The Nadine Murdoch granddaughter peel had significantly (*P* < 0.001) higher abundance of Sphingobacteriaceae and Chitinophagaceae than Royal Blue Murdoch. The Nadine Brunswick granddaughter peel had a higher relative abundance of Fimbriimonadaceae, Tepidisphaeraceae, Abditibacteriaceae, Alcaligenaceae and Cellvibrionaceae than Royal Blue Brunswick (Table S8).

The relative abundance of granddaughter tare soil bacterial communities was similar between cultivars, except Bacillaceae, Microbacteriaceae and Streptomycetaceae were significantly (*P* < 0.05) higher in Nadine, while Isosphaeraceae, and Planococcaceae were significantly (*P* < 0.05) higher in Royal Blue. Between the two fields, Bacillaceae, Microbacteriaceae, Streptomycetaceae, and Devosiaceae were significantly (*P* < 0.001) higher at Murdoch, while Xanthobacteraceae and Micrococcaceae were higher at Brunswick (Fig. S7).

### Horizontal Acquisition of Tuber Bacteria

**Fig. 2:**
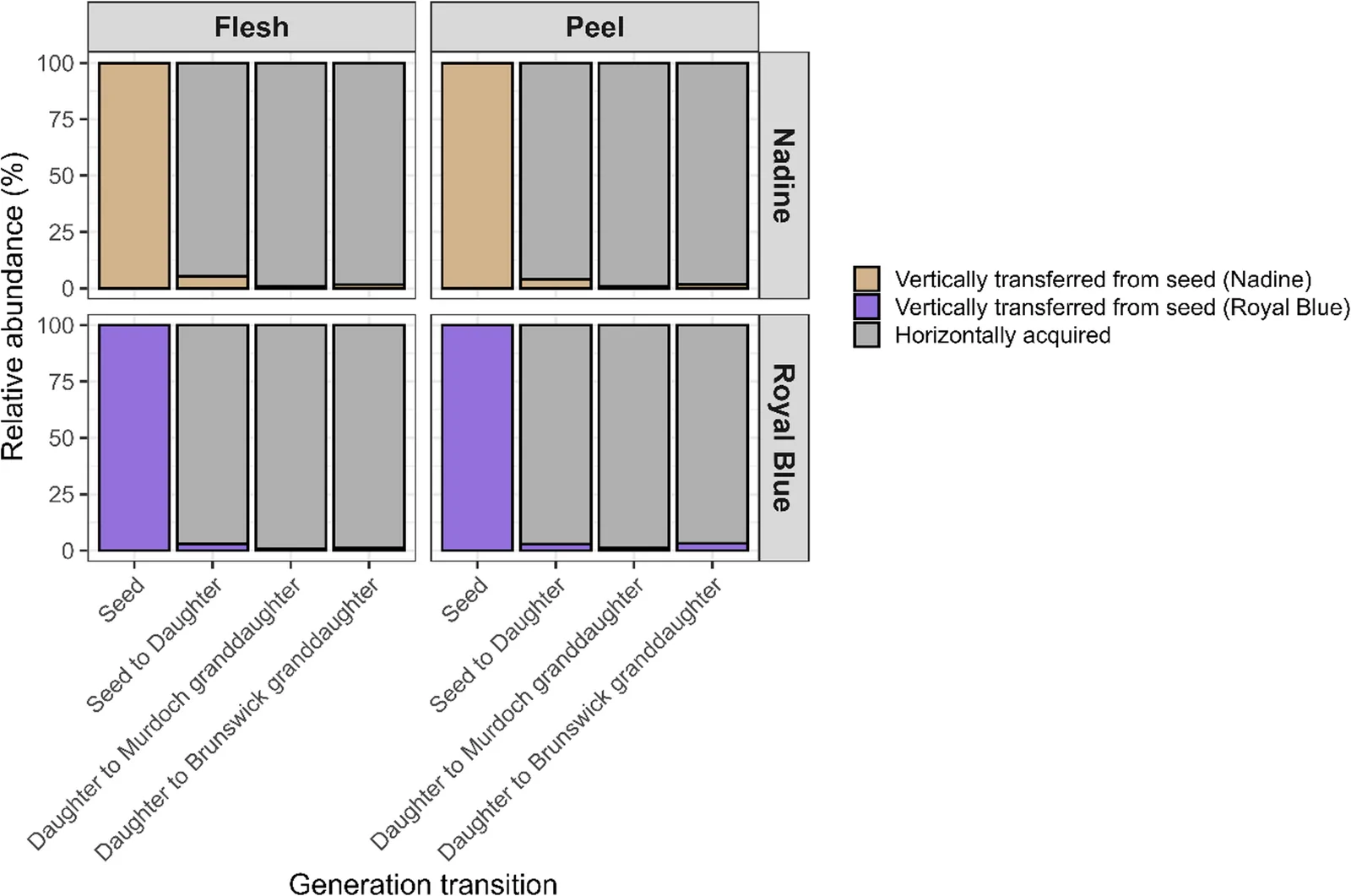
Relative abundance of vertically transferred or horizontally acquired ASVs in flesh and peel of Nadine and Royal Blue, from seed to daughter and daughter to granddaughter tubers, with seed tuber ASVs as the baseline for vertical transfer. Relative abundances were calculated using the total ASVs present in each generation

Horizontally acquired ASVs had significantly (*P* = 0.03) higher numbers than vertically transferred ASVs across all generation transitions, for both compartments and cultivars (Fig. 2). The number of horizontally acquired ASVs increased across generations in both cultivars, and was significantly higher (Wald χ²₁ = 812.5, *P* < 0.001) in granddaughter tubers than in daughter tubers. Peel had a significantly (*P* = 0.01) higher number of horizontally acquired ASVs than flesh. There were no significant differences (*P* > 0.05) between cultivars, with similar levels of horizontal ASV acquisition in both.

In Nadine, there were 327 ASVs in seed tubers, which increased to 3,488 in Murdoch granddaughter tubers. In Royal Blue, ASVs increased from 301 in seed tubers to 3,323 in Murdoch-grown granddaughter tubers (Fig. S8). Across cultivars, 118 ASVs differed between seed and daughter tubers, and more than 500 ASVs differed between daughter and granddaughter tubers in both fields (*P* < 0.001).

Higher numbers of unique ASVs were observed in Murdoch-grown granddaughters (1,955 peel and 537 flesh) than in Brunswick (793 peel and 738 flesh) (Fig. S9). Nadine’s granddaughters had 1,312 unique peel and 315 flesh ASVs at Murdoch, and 1,005 and 236 at Brunswick. Royal Blue had 1,337 peel and 337 flesh at Murdoch, and 282 peel and 959 flesh at Brunswick (Fig. S9). The largest overlap of ASVs was between the tare soil and peel (Fig. S9A and S9C). ASVs shared between the tare soil and flesh were low, and Royal Blue grown at Brunswick was the only sample with a high overlap (Fig. S9D).

The Murdoch granddaughter tuber peel had higher abundances (*P* < 0.001) of Xanthobacteraceae, Caulobacteraceae, and Flavobacteriaceae than the Brunswick granddaughters. Brunswick granddaughters had higher abundances (*P* < 0.05) of Pseudomonadaceae, Sphingobacteriaceae, and Enterobacteriaceae. Dominant tare soil ASVs were present in both peel and flesh, but were consistently higher in abundance in peel, indicating greater overlap between peel and tare soil than between tare soil and flesh (Figs. S5, S7 and S9).

### Vertical Transfer of Tuber Bacteria

Relative to the total ASVs of a given generation, vertical transfer was low and declined across generations (Fig. 2). After adjusting for field- and sample-level variation, the estimated probability of vertical transfer between generations was 1.8% (*P* < 0.001), with a 95% confidence interval of 1.3–2.4%. Vertical transfer was consistent in both the Brunswick and Murdoch fields, with negligible field variation. Vertical transfer probability was significantly (χ² = 17.48, *P* < 0.001) higher in flesh than in peel. This difference was similar (*P* = 0.286) for both seed-to-daughter and daughter-to-granddaughter transitions.

Cultivar effects were small but statistically significant (*P* = 0.034). In Nadine, seed-to-daughter vertical transfer was 5.29% in flesh and 3.89% in peel, and daughter-to-granddaughter values were 1.20% for flesh and 1.25% for peel. Only 37 ASVs (0.97%) were transferred across all three Nadine generations (Fig. S8A). The estimated probability of vertical transfer for Nadine was 1.8%.

Royal Blue followed the same generational and compartmental pattern, with seed-to-daughter vertical transfer of 3% in flesh and less than 2.8% in peel, and granddaughter values were 1.2% for flesh and 2.2% for peel. Only 29 ASVs (0.46%) were transferred across all three Royal Blue generations (Fig. S8B). The estimated probability of vertical transfer for Royal Blue was 1.5%, consistent across both fields.

**Fig. 3:**
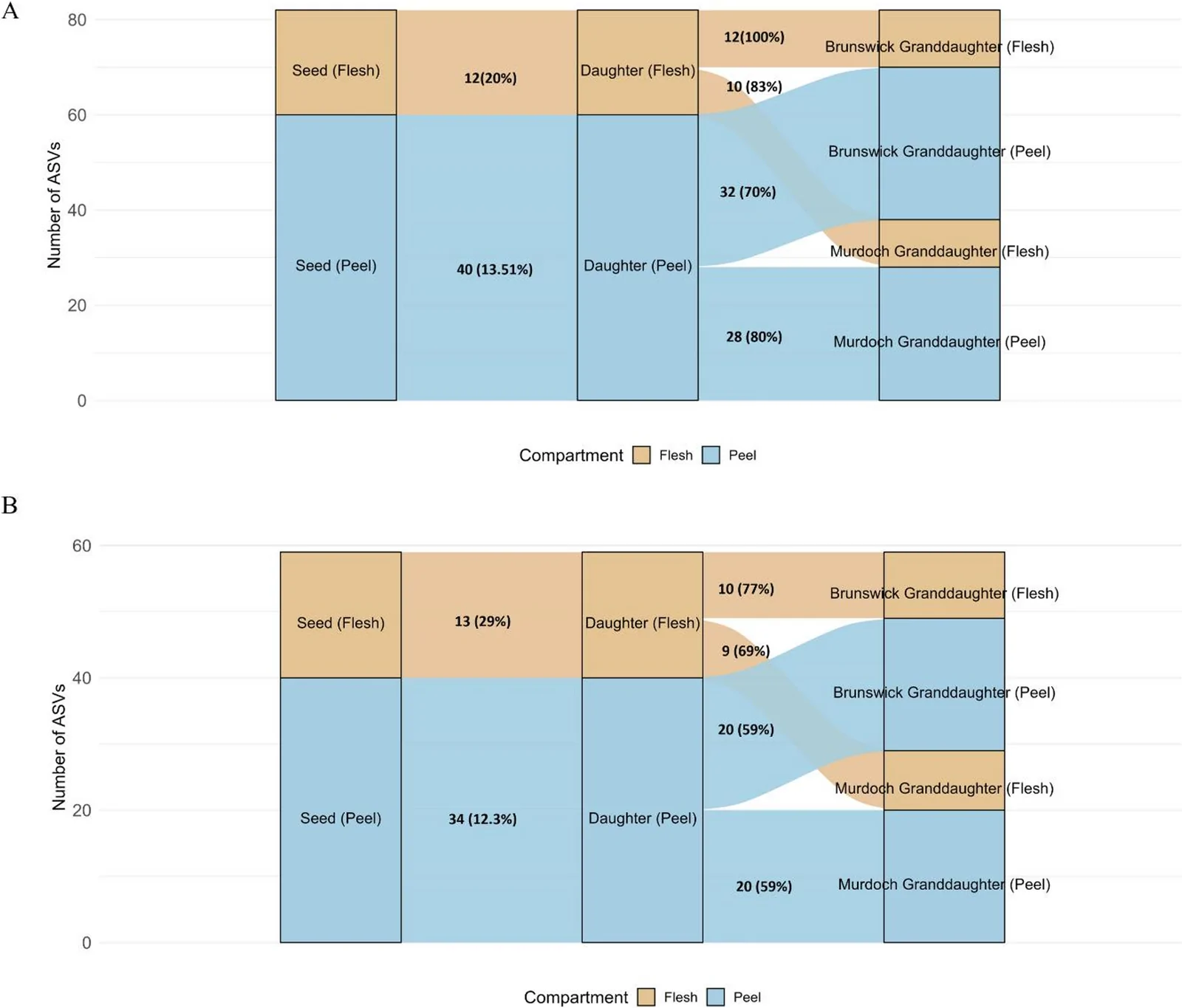
The proportion of ASVs transferred from potato seed tubers to daughter tubers and Brunswick and Murdoch granddaughter tubers in Nadine (**A**), and Royal Blue (**B**). The leftmost nodes represent ASVs identified in seed tubers, followed by those transferred to daughter tubers and those transferred to Murdoch or Brunswick granddaughter tubers. Percentages are the ASVs transferred at each stage relative to the previously transferred group

From the total ASVs in the seed tubers (peel and flesh), only 18% of ASVs present in the seed tubers were vertically transferred to the daughters. This was significantly (*P* < 0.05) higher than ASVs transferred from daughters to granddaughters, at 14% (Fig. 2). In Nadine, 52 seed tuber ASVs were vertically transferred to the daughter tubers, comprising 12 seed flesh-specific ASVs and 40 seed peel-specific ASVs. The 12 flesh ASVs were transferred to the Brunswick granddaughter flesh (100%) (Fig. 3A). Of the 40 peel ASVs, 32 were transferred to the Brunswick granddaughter peel (70%). In Royal Blue, 47 seed tuber ASVs were transferred to the daughter tubers, including 13 seed flesh-specific ASVs and 34 seed peel-specific ASVs. Of the 13 flesh ASVs, 10 were transferred to the Brunswick granddaughter’s flesh (77%). Of the peel ASVs, 20 were transferred to the Brunswick granddaughter peel (59%) (Fig. 3B).

**Fig. 4:**
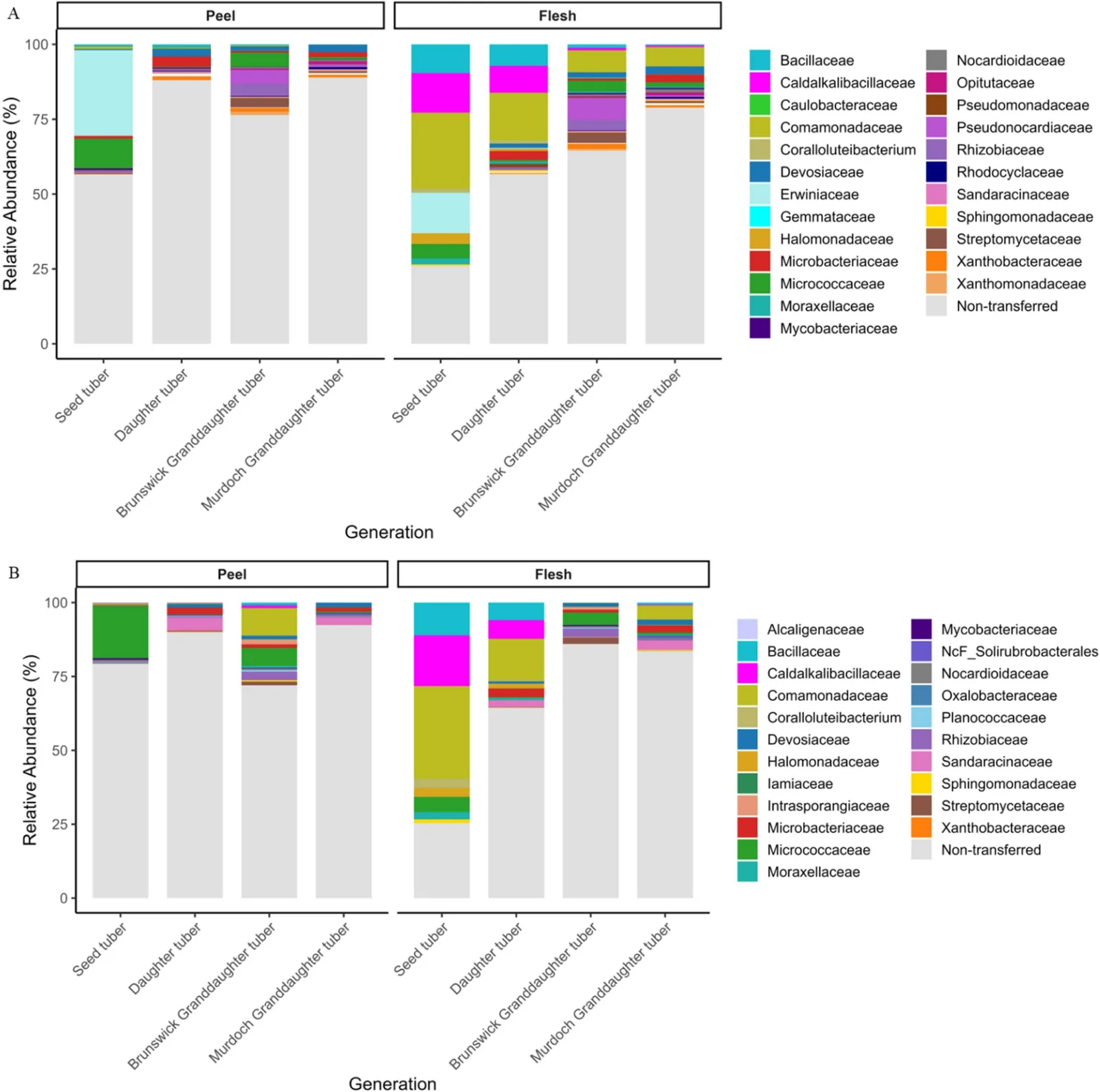
Distribution of vertically transferred ASVs in relation to the overall relative abundance, grouped by bacterial family across generations from seed to granddaughter in peel and flesh for cultivars Nadine (**A**) and Royal Blue (**B**). Each bar represents one cultivar–compartment–generation combination

Vertically transferred ASV families included both cultivar-specific and shared families (Fig. 4). The transferred ASVs belonged to 25 bacterial families in Nadine and 22 in Royal Blue. Of these, 16 families were common to both cultivars, including Devosiaceae, Microbacteriaceae, and Comamonadaceae in the peel, and Caldalkalibacillaceae in the flesh. *Sphingomonas koreensis* and *Caldalkalibacillus thermarum* were the only two species-level ASVs identified in all generations in both cultivars and both compartments. Within a cultivar, there was no significant difference (*P* > 0.33) in the relative abundance of any family transferred between generations.

### Stable Transfer of Bacteria

**Fig. 5:**
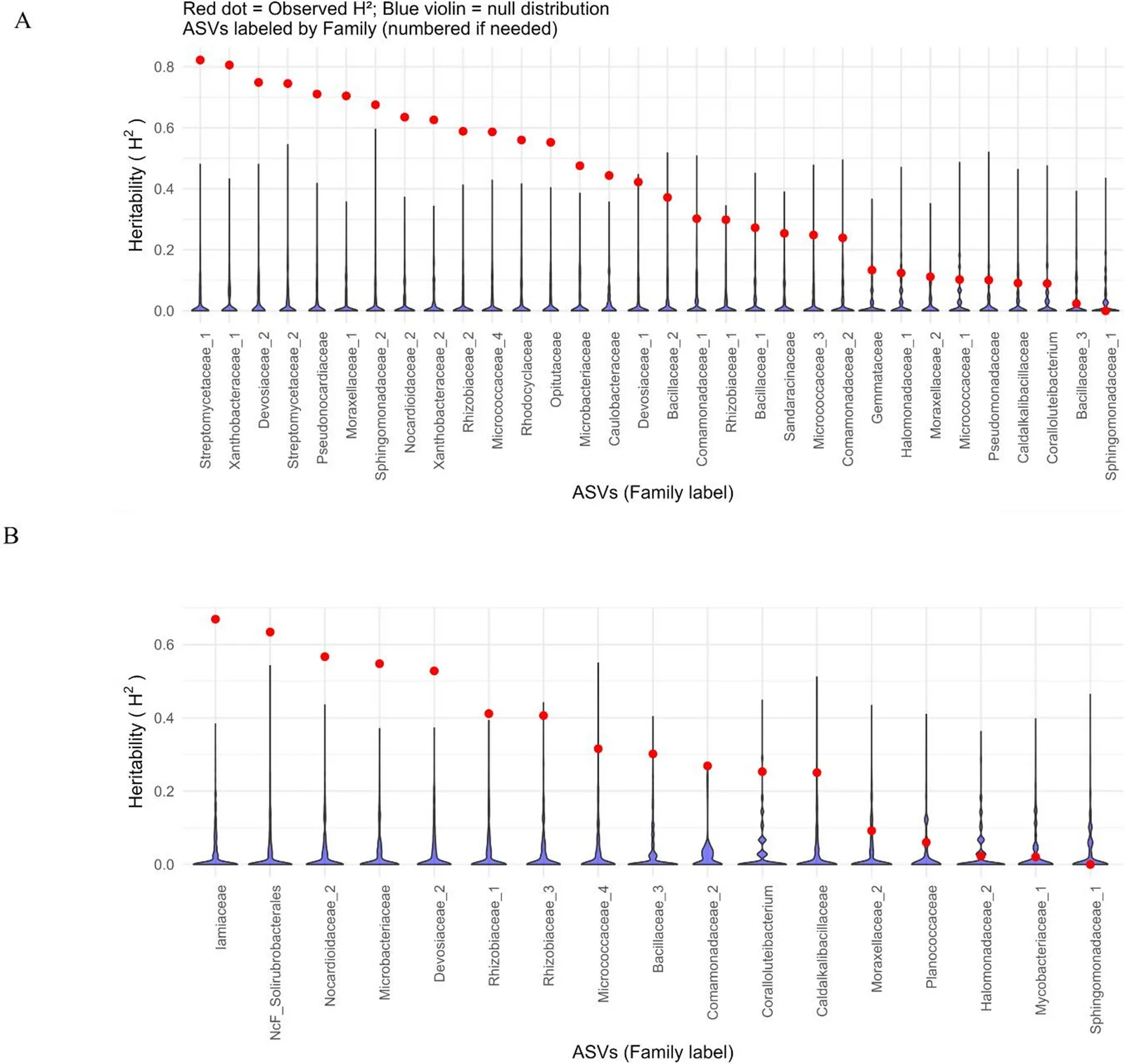
Broad-sense heritability (H²) of vertically transferred ASVs in Nadine (**A**) and Royal Blue (**B**). Observed H² values were overlaid as points, with red indicating statistical significance. Absolute ASV values were plotted and labelled by bacterial family only; numbering was used when multiple ASVs belonged to the same family

Vertically transmitted ASVs had H² values higher than expected by chance (*P* < 0.05), indicating stable abundance across generations. In Nadine, 64.9% of vertically transferred ASVs had significant generational consistency. The highest stability was found in Streptomycetaceae (H² = 0.82 and 0.74), Xanthobacteraceae (H² = 0.80 and 0.62), and Devosiaceae (H² = 0.74) (Fig. 5A). In Royal Blue, 58.6% of vertically transferred ASVs were consistent in abundance across generations. Moderate to high stability was observed in Iamiaceae (H² = 0.66), Caldalkalibacillaceae (H² = 0.50), Nocardioidaceae (H² = 0.56), and Bacillaceae (Fig. 5B). Overall H² values did not differ between cultivars (*P* = 0.4), indicating that observed differences reflected taxon identity rather than overall generational stability.

### Predicted Functions of Vertically Transferred Bacteria

Common predicted bacterial functions across cultivars and compartments included pathways such as amino acid metabolism and energy metabolism (Fig. S10). In Nadine, pathways related to secondary metabolite biosynthesis and vitamin metabolism were detected (Fig. S12A), whereas fermentation and compound degradation pathways were identified in Royal Blue (Fig. S12B). The peel community predicted functions were more diverse than the flesh community and were enriched in biosynthetic and energy pathways (e.g., glycolysis, lipid metabolism, and denitrification). The flesh ASVs enriched pathways related to catabolic and homeostatic functions and differed between cultivars. Stress-related functions, including denitrification and chorismate biosynthesis, were enriched in Nadine peel (Figs. S11 and S12A), while Royal Blue peel was enriched in oxidative energy metabolism pathways (Figs. S11 and S12B).

## Discussion

This study found that both vertical transfer and horizontal acquisition contributed to potato tuber bacterial communities. Horizontal acquisition dominated, particularly in the peel. However, a small and consistent subset of bacteria was vertically transferred from seed to daughter and granddaughter tubers, mainly in the flesh. These taxa had high H^2^ values across generations, indicating stable transfer. There were clear differences in the putative functions of transferred bacteria, likely reflecting differences in community composition across compartments and cultivars.

### Compartment, Cultivar and Field Communities

Potato peel and flesh bacterial communities differed in composition, supporting the hypothesis that tuber compartments host distinct microbiomes. Flesh communities were stable and low in diversity. Peel communities were more diverse and were similar to tare soil communities, reflecting exposure to soil. These compartment-specific patterns are consistent with previous reports of tissue-specific filtering in potato tubers [7, 36]. The compartment effect was stronger under field conditions than in controlled pot trials [7], suggesting tuber peel community assembly is primarily driven by horizontal acquisition [7, 8].

Significant cultivar–field interactions in the granddaughter generation suggest that different field soils supported different bacteria, and cultivars varied in their acquisition patterns. This variation is likely linked to cultivar-specific traits that influence the microbiome, such as root exudation profiles and tuber chemistry [37]. Several bacterial families were consistently associated with the peel, including beneficial bacteria from the Sandaracinaceae, Comamonadaceae, and Devosiaceae, suggesting a selective advantage. Some members of these families have known plant growth-promoting functions, such as *Variovorax* [38] and *Delftia* [39], and are commonly reported in soil attached to potato tubers [9].

### Stable Vertical Transfer and Predicted Roles of Transferred Taxa

Vertically transferred bacteria in potato were limited but consistent, particularly in tuber flesh. This indicates a selective process rather than a passive carryover of the tuber microbiome [40]. The limited vertical transfer in the field was consistent with a previous report on potatoes, which found higher transfer only in sterile or soil-free systems [8]. By considering all seed-associated bacteria across three generations, this study provides a direct estimate of vertical transfer, building on the field-unique ASVs reported by Song et al. (2024). This result supports the “microbial bottleneck” model described by Abdelfattah, Tack [6] and Rezki, Campion [41], in which only a small fraction of microbes was transferred across generations.

Similar observations have been reported in tomato [40, 42], wheat [43], and maize [35], where microbiome composition was largely driven by host genotype and environment, while a restricted set of taxa was vertically transferred. These similarities across crops suggest that selective vertical transfer is a common feature of plant-associated microbiomes, despite differences in host and propagation strategies [40]. The heritability of maize rhizosphere soil-associated bacteria was lower (H^2^ < 0.05–0.25) [35] than that observed in this study, likely reflecting variation in host compartment and experimental design, including sample size and variance structure, rather than habitat alone. In the current study, heritability was used as a measure of consistency of ASV abundance across generations within cultivars, not as evidence of host genetic control. The vegetative propagation of potato likely facilitates more direct bacterial transfer than rhizosphere soil, which is strongly influenced by environmental factors, and this may explain the relatively high H^2^ values observed. Vertical transfer was also confirmed at the species level for *Caldalkalibacillus thermarum* and *Sphingomonas koreensis*, but most ASVs lacked precise taxonomic resolution. Evidence from wheat and tomato indicates that only specific endophytic taxa are capable of stable vertical transfer [40, 43], while most seed-associated bacteria are transient, suggesting vertical transfer is selective.

The most likely mechanism of vertical transfer in potato is through internal movement within stolons and vascular tissues, enabling endophytic bacteria to pass from the mother plant to daughter tubers [7, 8, 44]. These bacteria likely persist in the tuber flesh with minimal exposure to the external environment, thereby supporting their survival during storage [8]. Upon sprouting, these bacteria colonise emerging shoots and act as the intial inoculum to establish microbial communities in the next generation. This process may help maintaintain a stable vertical transfer across successive generations of potatoes.

Bacterial taxa detected in potato tubers were previously reported in potato peel and flesh and were associated with plant growth and health [12, 45–47]. The highly stable Streptomycetaceae found in the present study were previously associated with potato vigour and were stable across generations [48], suggesting a potential link between vertical transfer and plant performance. Bacillaceae, including *Bacillus subtilis*, *B. amyloliquefaciens*, and *B. velezensis*, were reported to suppress disease in potato crops [12, 49, 50]. The consistent presence of Bacillaceae in both cultivars in the current study, and their predicted membrane-related pathways (e.g., CDP-diacylglycerol biosynthesis II, reported in *B. subtilis*), suggests a potential role in environmental stress tolerance. Similarly stable bacterial families previously associated with denitrification including Xanthobacteraceae (*Bradyrhizobium*), Xanthomonadaceae (*Rhodanobacter*), and Pseudomonadaceae (*Pseudomonas*) [51, 52], and Caldalkalibacillaceae (*Caldalkalibacillus thermarum*) which is heat-resistant [53], indicate that vertically transferred bacteria may have functions related to stress tolerance and host protection.

The stable members of Sphingomonadaceae (*Sphingomonas koreensis* and *Sphingopyxis*), Comamonadaceae (*Delftia* ), Micrococcaceae (e.g., *Arthrobacter*), Rhizobiaceae (*Rhizobium and Mesorhizobium*) and Pseudomonadaceae (*Pseudomonas*) detected in the present study have previously been linked to nutrient availability [54, 55], plant growth and biotic stress tolerance [12, 45, 47], in potatoes and other vegetatively propagated crops [13]. These taxa have also been consistently identified as endophytes in both culture-based [12, 56] and sequencing-based potato microbiome studies [13, 46, 57]. Their consistent detection and reported beneficial roles across independent studies suggest a functional role in potato.

Peel communities had greater predicted functional diversity than flesh communities, consistent with previous observations in soil-adjacent potato compartments [57–59]. However, the functional roles of these vertically transferred taxa cannot be confirmed without experimental validation. Cumulative evidence from studies of these taxa, including vertically transferred bacteria in tomato and other crops [38, 40, 41], suggests that these taxa are not random but are selectively maintained for plant growth promotion and protection. The taxa identified here therefore represent targets for further investigation of their potential roles in plant growth and stress tolerance, and should also be confirmed in other potato cultivars.

### Conclusion and Recommendations

This study assessed vertical transfer of bacterial communities across three generations of potato tubers in the flesh and peel. Communities differed between compartments, cultivars and fields, but a small but consistent group of bacteria potentially linked to important functions was transferred across generations, particularly in the flesh. Peel communities were more diverse and were acquired from the soil.

Future work should confirm vertical transfer of bacteria through experimental approaches such as culturomics and strain tracking, and extend sampling to more cultivars, generations, and environments. The functional roles of vertically transmitted bacteria should also be confirmed through metagenomic and transcriptomic approaches. Additional work is required to examine potato tuber fungal communities, to provide a more complete understanding of the potato microbiome and its heritability.

The results have implications for potato breeding programs and agronomic practices. Integrating vertically transferred bacteria with beneficial functions into breeding programs may reduce reliance on chemical fertilisers and increase crop resilience and productivity.

## Supplementary Information

Below is the link to the electronic supplementary material.

Supplementary Material 1.

## Data Availability

The sequencing data supporting the findings of this study have been submitted to the NCBI Sequence Read Archive (SRA) under BioProject accession number PRJNA1345547 and will be made publicly available upon publication.
