# Supplementary material for: Seed Potato Bacteria Transfer Across Generations Within the Tuber Flesh
Source: Microb Ecol. 2026 Apr 9;89:109. doi: 10.1007/s00248-026-02758-7 (PMC13171924; doi:10.1007/s00248-026-02758-7)
Supplement: Supplementary file 1 [file 248_2026_2758_MOESM1_ESM.docx]

**Supplementary Information - Seed potato bacteria transfer across generations within the tuber flesh**

**Table S1: Cultivars overview**

| **Characteristics** | **Nadine** | **Royal blue** |
| --- | --- | --- |
| **Variety used for (Fresh/process/seed)** | Fresh | Fresh |
| **Maturity after planting (days)** | 95-105 | 100-120 |
| **Heat susceptibility** | Resistant  [1] | Susceptible  [2] |
| **Plant height** | 30-50cm | 50-80 cm |
| **Country of origin** | The Netherlands | United Kingdom |
| **Leaf size, colour and flower colour** |  |  |
| **Tuber shape and colour** |  |  |

**Fig. S1** Production of three generations of Royal Blue and Nadine potatoes. The daughter tubers were cultivated in two separate fields.

**10m**

**Fig S2**: Schematic layout of a randomised experimental plot.

**Table S2. Primary nutrient composition of Wandalup compost**

| **Nutrient** | **% (dry weight)** | **Kg m⁻³ (as applied)** | **Kg tonne⁻¹ (as applied)** |
| --- | --- | --- | --- |
| Organic carbon (C) | 40.0 | 90 | 180 |
| Nitrogen (N) | 2.5 | 5.6 | 11.3 |
| Phosphorus (P) | 1.0 | 2.3 | 4.5 |
| Potassium (K) | 1.7 | 3.8 | 7.7 |
| Sulphur (S) | 1.7 | 3.8 | 7.7 |
| Calcium (Ca) | 5.4 | 12.2 | 24.3 |
| Magnesium (Mg) | 0.5 | 1.1 | 2.3 |
| Sodium (Na) | 0.4 | 0.9 | 1.8 |
| Iron (Fe) | 2000 | 450 | 900 |
| Manganese (Mn) | 350 | 79 | 158 |
| Boron (B) | 25 | 6 | 11 |
| Zinc (Zn) | 450 | 101 | 203 |
| Copper (Cu) | 100 | 23 | 45 |

**Physical and Chemical Properties of Wandalup Compost**

| **Metric** | **Value** |
| --- | --- |
| Organic Matter | 70% (dry weight) |
| Organic Carbon | 40% (dry weight) |
| Total Nitrogen | 2.2 % (dry weight) |
| C: N Ratio | 17:01 |
| Moisture Content | 60% (wet weight) |
| Bulk Density | 500kg/m2 |
| pH | 7.0 – 8.0 |
| Water Holding Capacity | 250% (dry weight) |
| Cation Exchange Capacity | 110 cmol/kg |

**Table S3: Physicochemical properties of soils collected from two planting sites**

| **Characteristics** | **Murdoch field** | **Brunswick field** |
| --- | --- | --- |
| Colour | Brown – Grey | Grey – Black |
| Gravel (%) | 5 | 0 |
| Texture | 2 (Loam) | 3.5 (Clay) |
| Ammonium Nitrogen (mg/Kg) | 2 | 3 |
| Nitrate Nitrogen (mg/Kg) | < 1 | 45 |
| Phosphorus Colwell(mg/Kg) | 15 | 289 |
| Potassium Colwell | 291 | 875 |
| Sulphur (mg/Kg) | 30.6 | 11.9 |
| Organic Carbon (%) | 1.52 | 4 |
| Conductivity (dS/m) | 0.237 | 0.173 |
| pH Level (CaCl2) | 6.9 | 6.1 |
| pH Level (H2O) | 7.3 | 7 |
| DTPA Copper (mg/Kg) | 0.90 | 2.06 |
| DTPA Iron (mg/Kg) | 22.90 | 255.90 |
| DTPA Manganese (mg/Kg) | 4.08 | 11.20 |
| DTPA Zinc (mg/Kg) | 7.62 | 10.40 |
| Exc. Aluminium (meq/100g) | 0.044 | 0.033 |
| Exc. Calcium (meq/100g) | 4.83 | 12.86 |
| Exc. Magnesium (meq/100g) | 0.90 | 3.29 |
| Exc. Potassium (meq/100g) | 0.84 | 2.12 |
| Exc. Sodium (meq/100g) | 0.40 | 0.28 |
| Boron Hot CaCl2 (mg/Kg) | 0.67 | 1.36 |

**Table S4: Nutrient composition of Red® and Prime® fertilisers**

| **Nutrient** | **Red®** | **Prime®** |
| --- | --- | --- |
| N (%) | 7.3 | 12 |
| P (%) | 8.1 | 5.2 |
| K (%) | 8.4 | 14 |
| S (%) | 4.1 | 6.2 |
| Ca (%) | 6.2 | 5.6 |
| Mg (%) | 1.5 | 1.2 |
| Fe (%) | 1 | 0.6 |
| Si (%) | 7.7 | 5.4 |
| C (%) | 3.2 | 1.1 |
| Zn (mg/Kg) | 1040 | 1023 |
| Mn (mg/Kg) | 793 | 721 |
| Cu (mg/Kg) | 308 | 312 |
| Mo (mg/Kg) | 8 | 14 |
| B (mg/Kg) | 104 | 102 |
| Co (mg/Kg) | 6 | 6 |
| Se (mg/Kg) | 0.6 | 1 |
| Bulk Density (Kg/m³) | 1020 | 1057 |

**Fig. S3** Tuber flesh samples were collected using horizontal and vertical cuts through internal tissues, including the cortex and outer and inner medulla.

**Methods**

**Statistical analyses**

Normalised ASVs were agglomerated to the family level using the tax_glom() function in phyloseq for taxonomic-level visualisation and filtering. Families with a median relative abundance >2% within any cultivar–generation group were retained for downstream analyses. To identify ASVs with significant differences in relative abundance across compartments, cultivars, or generations, a differential abundance analysis was performed using the DESeq2 package [3]. Raw ASV counts were used as input, and the standard DESeq2 workflow was applied, including normalisation and fitting of a negative binomial generalised linear model. ASVs with FDR-adjusted P < 0.05 were considered differentially abundant (Supplementary Tables S4 and S5).

DESeq2 was used to identify differentially abundant ASVs between compartments (peel vs. flesh), cultivars (Nadine vs. Royal Blue), and generations (seed vs. daughter and daughter vs. granddaughter) [3]. Pairwise cultivar comparisons within each generation were further assessed using Wilcoxon rank-sum tests. Significant differences were determined based on log₂ fold changes (Nadine/Royal Blue) and Benjamini–Hochberg adjusted P-values. Results were presented as heatmaps (using ggplot2) that displayed both effect sizes and statistical significance across generations and families.

The functional potential of bacterial communities was inferred using the Phylogenetic Investigation of Communities by Reconstruction of Unobserved States (PICRUSt2) pipeline [4]. Non-rarefied, decontaminated ASVs conserved across generations were used to preserve community structure and enable accurate prediction of gene family abundances [5]. Predicted functions were categorised into KEGG Orthology (KO) groups, Enzyme Commission (EC) numbers, and MetaCyc metabolic pathways. KEGG Orthology identifiers were mapped to KEGG pathways using KEGG Mapper to identify higher-order functional associations. The pathways were grouped into broad functional categories (e.g., amino acid metabolism, energy metabolism, lipid metabolism) and further resolved into specific sub-pathways. Pathway abundances were normalised using Counts Per Million (CPM) [6], and the relative abundances of each pathway were visualised as stacked bar plots using the ggplot2 package. Only the top five most abundant specific pathways within each broad functional category and the top eight broad categories were displayed. Differential abundance between peel and flesh was tested separately for each cultivar using Wilcoxon rank-sum tests. Peel was used as the reference group. Positive log₂ fold changes indicate higher abundance in peel, and negative values indicate higher abundance in flesh. P values were corrected using the Benjamini–Hochberg false discovery rate procedure, and pathways with FDR-adjusted P < 0.05 were considered significant. Effect sizes were summarised as log₂ fold change and the median relative abundance in each compartment. The top twenty significant pathways for each compartment and cultivar, as well as the top twenty significant pathways overall, were visualised using heatmaps generated with the pheatmap package.

**Table S5: Mean richness, Shannon diversity and evenness (±Standard Error) for each cultivar, generation and compartment (flesh and peel)**

| **Cultivar** | **Generation** | **Compartment** | **Mean_**  **Richness** | **SE_**  **Richness** | **Mean_**  **Shannon** | **SE_**  **Shannon** | **Mean_**  **Evenness** | **SE_**  **Evenness** |
| --- | --- | --- | --- | --- | --- | --- | --- | --- |
| Nadine | Seed tuber | Flesh | 12 | 1.61 | 1.95 | 0.125 | 0.81 | 0.03 |
| Nadine | Seed tuber | Peel | 53.8 | 6.71 | 2.52 | 0.147 | 0.65 | 0.04 |
| Nadine | Daughter tuber | Flesh | 31.7 | 3.90 | 2.95 | 0.150 | 0.87 | 0.02 |
| Nadine | Daughter tuber | Peel | 209.6 | 8.39 | 4.39 | 0.080 | 0.82 | 0.02 |
| Nadine | Brunswick Granddaughter tuber | Flesh | 136 | 20.95 | 4.11 | 0.151 | 0.86 | 0.01 |
| Nadine | Brunswick Granddaughter tuber | Peel | 489 | 33.30 | 5.08 | 0.107 | 0.82 | 0.01 |
| Nadine | Murdoch Granddaughter tuber | Flesh | 185 | 18.26 | 4.65 | 0.096 | 0.90 | 0.00 |
| Nadine | Murdoch Granddaughter tuber | Peel | 687.8 | 26.08 | 5.66 | 0.048 | 0.87 | 0.01 |
| Royal Blue | Seed tuber | Flesh | 10.9 | 0.96 | 2.00 | 0.094 | 0.85 | 0.01 |
| Royal Blue | Seed tuber | Peel | 56.7 | 4.18 | 2.68 | 0.073 | 0.67 | 0.01 |
| Royal Blue | Daughter tuber | Flesh | 57.4 | 18.20 | 3.22 | 0.267 | 0.87 | 0.02 |
| Royal Blue | Daughter tuber | Peel | 232.3 | 18.80 | 4.44 | 0.121 | 0.82 | 0.02 |
| Royal Blue | Brunswick Granddaughter tuber | Flesh | 457.3 | 31.46 | 4.91 | 0.122 | 0.80 | 0.01 |
| Royal Blue | Brunswick Granddaughter tuber | Peel | 112.2 | 7.81 | 4.07 | 0.094 | 0.87 | 0.01 |
| Royal Blue | Murdoch Granddaughter tuber | Flesh | 194.2 | 12.75 | 4.68 | 0.062 | 0.89 | 0.01 |
| Royal Blue | Murdoch Granddaughter tuber | Peel | 682.6 | 24.67 | 5.52 | 0.078 | 0.85 | 0.01 |

**Table S6: Generation-wise pairwise contrasts of Shannon diversity in peel and flesh for Nadine and Royal Blue.**

| **Cultivar** | **Compartment** | **Comparison** | **P_value** | **Significance** |
| --- | --- | --- | --- | --- |
| Nadine | Peel | Seed tuber vs Daughter tuber | <0.0001 | **** |
| Nadine | Peel | Seed tuber vs Brunswick Granddaughter tuber | <0.0001 | **** |
| Nadine | Peel | Seed tuber vs Murdoch Granddaughter tuber | <0.0001 | **** |
| Nadine | Peel | Daughter tuber vs Brunswick Granddaughter tuber | <0.0001 | *** |
| Nadine | Peel | Daughter tuber vs Murdoch Granddaughter tuber | <0.0001 | **** |
| Nadine | Peel | Brunswick Granddaughter tuber vs Murdoch Granddaughter tuber | <0.001 | ** |
| Nadine | Flesh | Seed tuber vs Daughter tuber | <0.0001 | **** |
| Nadine | Flesh | Seed tuber vs Brunswick Granddaughter tuber | <0.0001 | **** |
| Nadine | Flesh | Seed tuber vs Murdoch Granddaughter tuber | <0.0001 | **** |
| Nadine | Flesh | Daughter tuber vs Brunswick Granddaughter tuber | <0.0001 | **** |
| Nadine | Flesh | Daughter tuber vs Murdoch Granddaughter tuber | <0.0001 | **** |
| Nadine | Flesh | Brunswick Granddaughter tuber vs Murdoch Granddaughter tuber | <0.0326 | * |
| Royal Blue | Peel | Seed tuber vs Daughter tuber | <0.0001 | **** |
| Royal Blue | Peel | Seed tuber vs Brunswick Granddaughter tuber | <0.0001 | **** |
| Royal Blue | Peel | Seed tuber vs Murdoch Granddaughter tuber | <0.0001 | **** |
| Royal Blue | Peel | Daughter tuber vs Brunswick Granddaughter tuber | <0.0369 | * |
| Royal Blue | Peel | Daughter tuber vs Murdoch Granddaughter tuber | <0.0001 | **** |
| Royal Blue | Peel | Brunswick Granddaughter tuber vs Murdoch Granddaughter tuber | <0.0001 | **** |
| Royal Blue | Flesh | Seed tuber vs Daughter tuber | <0.0001 | **** |
| Royal Blue | Flesh | Seed tuber vs Brunswick Granddaughter tuber | <0.0001 | **** |
| Royal Blue | Flesh | Seed tuber vs Murdoch Granddaughter tuber | <0.0001 | **** |
| Royal Blue | Flesh | Daughter tuber vs Brunswick Granddaughter tuber | <0.0001 | **** |
| Royal Blue | Flesh | Daughter tuber vs Murdoch Granddaughter tuber | <0.0001 | **** |
| Royal Blue | Flesh | Brunswick Granddaughter tuber vs Murdoch Granddaughter tuber | 0.715 | Ns |

**Table S7. Top statistically differentially abundant bacterial families in the potato flesh microbiome of Nadine and Royal Blue cultivars.**

| **Family** | **Generation** | **Nadine_median** | **RoyalBlue_median** | **Log2FC_NvRB** | **Direction** | **p_value** | **Significance** |
| --- | --- | --- | --- | --- | --- | --- | --- |
| Caldalkalibacillaceae | Brunswick Granddaughter tuber | 0.73 | 0.01 | 5.99 | Nadine | 0.000173 | *** |
| Bacillaceae | Brunswick Granddaughter tuber | 1.49 | 0.21 | 2.82 | Nadine | 0.000487 | *** |
| 211ds20 | Murdoch Granddaughter tuber | 0 | 0.73 | -16.16 | Royal Blue | 0.00101 | ** |
| Cellvibrionaceae | Brunswick Granddaughter tuber | 1.93 | 0.17 | 3.5 | Nadine | 0.00249 | ** |
| Methylophilaceae | Brunswick Granddaughter tuber | 0.47 | 1.92 | -2.02 | Royal Blue | 0.00279 | ** |
| Micropepsaceae | Brunswick Granddaughter tuber | 0 | 0.04 | -11.8 | Royal Blue | 0.00364 | ** |
| Sandaracinaceae | Murdoch Granddaughter tuber | 3.18 | 12.06 | -1.92 | Royal Blue | 0.0052 | ** |
| Bacteriovoracaceae | Brunswick Granddaughter tuber | 0 | 0.02 | -10.94 | Royal Blue | 0.00597 | ** |
| Steroidobacteraceae | Brunswick Granddaughter tuber | 0 | 0.01 | -9.43 | Royal Blue | 0.00597 | ** |
| Solirubrobacteraceae | Murdoch Granddaughter tuber | 0.92 | 0.28 | 1.7 | Nadine | 0.00908 | ** |
| Polyangiaceae | Murdoch Granddaughter tuber | 0.16 | 0 | 13.93 | Nadine | 0.01 | * |
| Rhizobiaceae | Brunswick Granddaughter tuber | 6.26 | 9.02 | -0.53 | Royal Blue | 0.0147 | * |
| Silvanigrellaceae | Brunswick Granddaughter tuber | 0 | 0 | -8.06 | Royal Blue | 0.0149 | * |
| Vermiphilaceae | Brunswick Granddaughter tuber | 0 | 0.01 | -9.06 | Royal Blue | 0.0149 | * |
| NS9 marine group | Murdoch Granddaughter tuber | 0 | 0.15 | -13.87 | Royal Blue | 0.0156 | * |
| Rubinisphaeraceae | Brunswick Granddaughter tuber | 0 | 0.07 | -12.7 | Royal Blue | 0.0172 | * |
| Pseudonocardiaceae | Murdoch Granddaughter tuber | 1.82 | 0.36 | 2.35 | Nadine | 0.0207 | * |
| Ktedonobacteraceae | Brunswick Granddaughter tuber | 0 | 0.03 | -11.64 | Royal Blue | 0.0212 | * |
| Thermomonosporaceae | Brunswick Granddaughter tuber | 0 | 0.04 | -12.02 | Royal Blue | 0.0212 | * |
| Mycobacteriaceae | Murdoch Granddaughter tuber | 0.71 | 0.36 | 0.97 | Nadine | 0.0228 | * |

**Fig. S4** The relative abundance (%) of dominant bacterial families in the flesh of Nadine and Royal Blue cultivars across tuber generations (Seed, Daughter, and Granddaughter tubers from two sites). Only families with a median abundance >2% in at least one group are shown.

**Fig. S5** Comparison of the bacterial family composition and differential abundance in potato peel bacteria. A stacked bar plot shows the relative abundance (%) of dominant bacterial families in the Peel of Nadine and Royal Blue cultivars across tuber generations (Seed, Daughter and Granddaughter tubers from two sites). Only families with a median abundance >2% in at least one group.

**Fig. S6** Differential abundance of bacterial families between potato cultivars Nadine and Royal Blue across peel and flesh compartments. Bars represent the base-2 logarithm of fold change (log₂ fold change) in median relative abundance between cultivars (Nadine / Royal Blue) for each bacterial family. Positive values indicate enrichment in Nadine, and negative values indicate enrichment in Royal Blue. Asterisks denote statistical significance based on Wilcoxon rank-sum tests: P < 0.05 (*), P< 0.01 (**), P < 0.001 (***). Only families with significant differences (P < 0.05) in at least one compartment are shown.

**Table S8. Top 20 differentially abundant bacterial families in the potato peel microbiome of Nadine and Royal Blue cultivars**

| **Family** | **Generation** | **Nadine_median** | **RoyalBlue_median** | **Log2FC_NvRB** | **Direction** | **p_value** | **Significance** |
| --- | --- | --- | --- | --- | --- | --- | --- |
| Comamonadaceae | Brunswick Granddaughter tuber | 6.1 | 14.18 | -1.22 | Royal Blue | 1.08E-05 | *** |
| Sphingobacteriaceae | Murdoch Granddaughter tuber | 1.2 | 0.31 | 1.95 | Nadine | 2.17E-05 | *** |
| Bacillaceae | Brunswick Granddaughter tuber | 0.24 | 1.65 | -2.76 | Royal Blue | 7.58E-05 | *** |
| Fimbriimonadaceae | Brunswick Granddaughter tuber | 0.39 | 0.07 | 2.52 | Nadine | 0.000529 | *** |
| Tepidisphaeraceae | Brunswick Granddaughter tuber | 0.17 | 0 | 14.07 | Nadine | 0.00068 | *** |
| Abditibacteriaceae | Brunswick Granddaughter tuber | 0.02 | 0 | 10.88 | Nadine | 0.000751 | *** |
| Alcaligenaceae | Brunswick Granddaughter tuber | 0.03 | 0 | 11.49 | Nadine | 0.000751 | *** |
| Cellvibrionaceae | Brunswick Granddaughter tuber | 1.73 | 0.19 | 3.19 | Nadine | 0.00099 | *** |
| Caldalkalibacillaceae | Brunswick Granddaughter tuber | 0 | 0.91 | -16.47 | Royal Blue | 0.00103 | ** |
| Chitinophagaceae | Murdoch Granddaughter tuber | 4.1 | 2.74 | 0.58 | Nadine | 0.00105 | ** |
| Moraxellaceae | Brunswick Granddaughter tuber | 0.56 | 1.67 | -1.57 | Royal Blue | 0.00105 | ** |
| Geodermatophilaceae | Brunswick Granddaughter tuber | 0.13 | 0 | 13.61 | Nadine | 0.00133 | ** |
| Yersiniaceae | Seed tuber | 0 | 0.98 | -16.58 | Royal Blue | 0.00192 | ** |
| Comamonadaceae | Daughter tuber | 2.11 | 3.88 | -0.88 | Royal Blue | 0.00209 | ** |
| Dermacoccaceae | Seed tuber | 2.25 | 13.25 | -2.56 | Royal Blue | 0.00209 | ** |
| Sandaracinaceae | Murdoch Granddaughter tuber | 3.15 | 14.51 | -2.21 | Royal Blue | 0.00209 | ** |
| Cellulomonadaceae | Brunswick Granddaughter tuber | 0.09 | 0 | 13.06 | Nadine | 0.00221 | ** |
| Cyclobacteriaceae | Brunswick Granddaughter tuber | 0.01 | 0 | 10.41 | Nadine | 0.00221 | ** |
| Rubinisphaeraceae | Brunswick Granddaughter tuber | 0.05 | 0 | 12.28 | Nadine | 0.00221 | ** |
| Geodermatophilaceae | Murdoch Granddaughter tuber | 0.07 | 0 | 12.81 | Nadine | 0.00382 | ** |

**Fig. S7** Relative abundance of tare soil bacterial families in Nadine and Royal Blue granddaughters from two fields.

**Fig. S8** Venn diagrams showing ASV overlap across seed, daughter, and granddaughter tubers for Nadine (A) and Royal Blue (B) cultivars.

**Fig. S9** ASV overlap among peel, flesh, and tare soil compartments in cv. Nadine (Murdoch, A; Brunswick, B) and Royal Blue granddaughter tubers (Murdoch, C; Brunswick, D). Each Venn diagram illustrates ASVs that are unique to individual compartments, and those shared between two or all three compartments.

**Fig. S10** Predicted functional profiles of conserved ASVs in Nadine (A) and Royal Blue (B) tubers. The stacked bar graph shows the top five KEGG pathways within the eight major metabolic categories predicted from conserved ASVs in Nadine. Broad categories shared by both cultivars are labelled with the same text colour.

**Fig. S11** Compartment-specific functional profiles of conserved bacteria in potato tubers. (A) Nadine: Top 20 statistically enriched pathways between peel and flesh. (B) Royal Blue: Top 20 statistically enriched pathways between peel and flesh using the same statistical threshold. Positive values indicate enrichment in the peel, while negative values indicate enrichment in the flesh. Only pathways with adjusted p < 0.05 are shown.

**Fig. S12** Top 20 statistically significant KEGG pathways predicted from vertically transferred bacterial ASVs across peel and flesh compartments in potato tubers. A Nadine; B: Royal Blue. Colours represent log₂-transformed median Counts Per Million values, scaled from 0 to 10, reflecting relative abundance within each compartment.

**References**

1. McPharlin I, Learmonth S, Shimmin T, Tooke D, Dawson P. Helping the Western Australian potato industry capture winter production opportunities in the Mid-West. Hort Australia Ltd. 2013. <https://ausveg.com.au/app/data/technical-insights/docs/PT13009.PDF>
2. Obiero CO, Milroy SP, Bell RW. Increasing frequency of high-temperature episodes in potato growing regions of Western Australia and its impacts on plant and tuber growth. Arch Agron Soil Sci. 2021;68(14):1988–2004. <https://doi.org/10.1080/03650340.2021.1948018>
3. Abegaz F, Abedini D, White F, Guerrieri A, Zancarini A, Dong L, Westerhuis JA, van Eeuwijk F, Bouwmeester H, Smilde AK. A strategy for differential abundance analysis of sparse microbiome data with group-wise structured zeros. Sci Rep. 2024;14(1):12433. <https://doi.org/10.1038/s41598-024-62437-w>
4. Douglas GM, Maffei VJ, Zaneveld JR, Yurgel SN, Brown JR, Taylor CM, Huttenhower C, Langille MGI. PICRUSt2 for prediction of metagenome functions. Nat Biotechnol. 2020;38(6):685–688. <https://doi.org/10.1038/s41587-020-0548-6>
5. Davis NM, Proctor DM, Holmes SP, Relman DA, Callahan BJ. Simple statistical identification and removal of contaminant sequences in marker-gene and metagenomics data. Microbiome. 2018;6(1):226. <https://doi.org/10.1186/s40168-018-0605-2>
6. Robinson MD, McCarthy DJ, Smyth GK. edgeR: a Bioconductor package for differential expression analysis of digital gene expression data. Bioinformatics. 2010;26(1):139–140. <https://doi.org/10.1093/bioinformatics/btp616>
